# Squamous Cell Carcinoma of the Bladder Mimicking Interstitial Cystitis and Voiding Dysfunction

**DOI:** 10.1155/2013/924918

**Published:** 2013-10-10

**Authors:** Colton Prudnick, Chad Morley, Robert Shapiro, Stanley Zaslau

**Affiliations:** ^1^West Virginia University School of Medicine, P.O. Box 9238, Morgantown, WV 26506, USA; ^2^Division of Urology, West Virginia University School of Medicine, P.O. Box 9238, Morgantown, WV 26506, USA; ^3^Department of Obstetrics and Gynecology, West Virginia University School of Medicine, P.O. Box 9238, Morgantown, WV 26506, USA

## Abstract

Squamous cell carcinoma (SCC) of the bladder is a relatively uncommon cause of bladder cancer accounting for <5% of bladder tumors in the western countries. SCC has a slight male predominance and tends to occur in the seventh decade of life. The main presenting symptom of SCC is hematuria, and development of this tumor in the western world is associated most closely with chronic indwelling catheters and spinal cord injuries. A 39-year-old Caucasian female presented with bladder and lower abdominal pain, urinary frequency, and nocturia which was originally believed to be interstitial cystitis (IC) but was later diagnosed as SCC of the bladder. Presentation of SCC without hematuria is an uncommon presentation, but the absence of this symptom should not lead a practitioner to exclude the diagnosis of SCC. This case is being reported in an attempt to explain the delay and difficulty of diagnosis. Background on the risk factors for SCC of the bladder and the typical presenting symptoms of bladder SCC and IC are also reviewed.

## 1. Introduction

In western regions, primary SCC of the bladder is uncommon with an incidence of 1.2–4.5% of all vesical tumors [1–6]. There is a slightly greater male-to-female predominance ranging from 1.25 : 1 to 1.8 : 1 for the disease, and it occurs most frequently in the seventh decade of life [6]. Several risk factors for squamous cell carcinoma of the bladder include cigarette smoking, chronic recurrent urinary tract infections (UTIs), schistosomiasis secondary to *Schistosoma haematobium* infection, urinary tract calculi [6–9], clean intermittent self-catheterization [10–12], long-term catheterization, and a neurogenic bladder in spinal cord injured patients [7–9]. In contrast to bladder SCC, IC sufferers tend to be female and predominantly middle-aged [13]. Typically, presenting symptoms consist of subacute development of pain on bladder filling, urinary frequency unrelieved at night, urgency, and frequency of micturition [13, 14]. Over time, patients will complain of varying degrees of symptoms without total relief at any time despite antibiotic treatment. Urine cultures are negative and some urethral/vaginal tenderness may be the only physical exam finding [13]. When present in male patients, the most common symptoms are analogous to those in females. These include suprapubic pain, urinary frequency, and dysuria [15].

## 2. Case Presentation

The patient was a 39-year-old white female who had a 3-year history of bladder and lower abdominal pain. Additional symptoms were urinary frequency, nocturia, a weak force of stream, and straining to void. She had been inconsistently performing clean intermittent catheterization (CIC) for elevated postvoid residual. Previous urine cytology had been negative for malignancy. The patient underwent diagnostic cystoscopy and urinalysis for her symptoms. The urinalysis revealed a moderate amount of leukocytes and the presence of nitrites. Cystoscopy showed chronic inflammatory changes in the bladder but no definite tumors or stones. Following the procedure, the patient reported an improvement in both her urethral pain and urinary urgency. The procedural findings and symptomatology changes led her practitioners to surmise that she most likely had interstitial cystitis/urethral syndrome. Consequently, she was diagnosed as having pelvic pain and nonobstructive incomplete bladder emptying. The patient was treated with Ciprofloxacin, Elmiron, and Cysta Q and was instructed to consistently self-catheterize twice daily. 

Approximately 5 months later, the patient presented to the emergency department for increased pelvic pain. The pain was believed to be an exacerbation of her cystitis and she was admitted to the urology service from the emergency room. During her admission, the patient continued to have persistent fever despite appropriate antibiotic therapy, and therefore a CT scan and ultrasound studies were ordered and showed a thickened bladder and some obstruction of the ureters. She subsequently underwent cystourethroscopy and biopsy of the bladder. Cystourethroscopy findings included a large tailgating mass from the bladder dome and wool like irregularity in the lateral walls of the bladder. Pathological findings of the biopsies revealed invasive moderately differentiated keratinizing SCC involving the full thickness of the bladder wall and adjacent soft tissue. Metastatic carcinoma was also found in the right-sided pelvic lymph nodes. Gynecology oncology was consulted to determine if there was cervical involvement. The cervix was found to be normal to gross inspection and without obvious lesions following a loop electrosurgical excision procedure (LEEP). The following week, the patient underwent radical cystectomy with ileal conduit urinary diversion, radical hysterectomy, and right-sided pelvic lymphadenectomy. The patient started with chemotherapy. Unfortunately she failed in all attempts at treatment and expired 6 months later.

## 3. Discussion

In multiple studies, hematuria was the principal presenting symptom of SCC of the bladder with a prevalence of 63–100% followed by irritative bladder symptoms with a prevalence of 33–67% [6–11]. Several other studies also reported lower abdominal pain as a common presenting symptom of SCC of the bladder. Kaye et al. reported a case in which a patient presented with gross hematuria [12]. In a case series documented by Pattison et al., the presenting symptoms of four patients with SCC of the bladder included microscopic hematuria, suprapubic pain, and continuing UTIs [16]. A case reported by Zaidi et al. described a woman who was diagnosed with bladder SCC following investigation of hematuria [11]. Serretta et al. described the main symptoms of dysuria and hematuria in a group of IC patients who were later diagnosed with bladder SCC [3]. In the 22 patients in Wang et al.'s retrospective study on SCC of the bladder, hematuria was the most common symptom followed by irritation of the bladder [17]. Finally, in a patient who was later diagnosed with *Schistosoma* induced SCC of the bladder, presenting symptoms were colicky pain in the abdomen associated with micturition and low backache [18]. 

In the present case, the patient was believed to have IC because the original clinical presentation and work up more closely resembled IC than bladder SCC. Epidemiologically, the symptoms presented by this patient were misleading and therefore impeded an accurate diagnosis. As a white, middle-aged female, she was much more likely to have IC than SCC of the bladder given her symptoms, exam, cystoscopy, and demographic. Furthermore, despite the fact that bladder and lower abdominal pain are presenting symptoms for both IC and bladder SCC, the presence of some symptoms (urinary frequency and nocturia) and the absence of others (hematuria) lent greater credence to the diagnosis of IC. Her original urine cytology and cystoscopy also diverted attention from the diagnosis of bladder SCC and suggested IC. Although her clinical presentation was more similar to that of IC, the patient did have risk factors for bladder SCC: a history of clean intermittent catheterization and a 60-pack-year smoking history. 

Since the diagnosis of IC largely relies on the exclusion of other bladder conditions [19], there is great need for serum and/or urinary biomarkers to aid in the diagnostic process. Recent studies are encouraging in which potential IC biomarkers have been identified in the urine of patients, suggesting that the potential for noninvasive and objective methods of diagnosis may be possible [20, 21]. Further studies on these biomarkers may allow for a more confident and less subjective diagnosis.

## 4. Conclusion

This patient's bladder SCC masqueraded as IC and voiding dysfunction. Some researchers propose that to overcome such clinical subterfuge and accurately diagnose bladder SCC, a high index of suspicion of bladder SCC should be maintained in patients who have certain risk factors for bladder SCC, which include UTIs in patients using clean intermittent self-catheterization (CISC) [13]. Kaye et al. feel that measures including cystoscopy should be performed for patients undergoing intermittent self-catheterization who suffer from hematuria [12]. However, Fletcher believes that any unexplained bladder symptoms should be investigated with cystoscopy and biopsies and that these measures should be offered annually to all such patients after 5 years of CISC [13]. Furthermore, Fletcher recommends that the frequency of cystoscopy be increased if keratinizing squamous metaplasia is identified [13]. Lastly, the pending addition of APF as a biomarker for IC would increase diagnostic confidence and potentially help to avoid cases such as this one in the future [20, 21].
